# Evaluation of different methods to obtain primary mammary epithelial cell cultures from canine spontaneous mammary gland tumors

**DOI:** 10.1186/1753-6561-7-S2-P41

**Published:** 2013-04-04

**Authors:** Luciana B Gentile, Márcia K Nagamine, Isis P Jesus, Fábio T Toyota, Adriana T Nishiya, Tatiane M Giovani, Daniel S Sanches, Heidge Fukumasu, Maria Lúcia Z Dagli

**Affiliations:** 1 Laboratory of Comparative and Experimental Oncology, Department of Pathology, School of Veterinary Medicine and Animal Sciences, University of São Paulo (USP), São Paulo, Brazil; 2 Veterinary Hospital Cães e Gatos, Osasco, São Paulo, Brazil; 3 University Anhembi Morumbi, São Paulo, Brazil; 4 Department of Pathology, School of Veterinary Medicine and Animal Sciences, University of São Paulo (USP), São Paulo, Brazil

## Background

Mammary gland neoplasms are the most prevalent tumors in dogs, and their treatment is still challenging. A crucial problem in the handling of this type of neoplasm is to obtain primary mammary epithelial cell cultures from the original tumors. The aim of this study was to determine the best conditions to culture primary mammary epithelial cells from several histological types of canine breast tumors.

## Materials and methods

Several culturing conditions have been tested including enzymes such as collagenases types III and IV, trypsin and hyaluronidase, differential centrifugation and trypsinization. Four tumor samples were processed to obtain organoids, stromal and epithelial cells. The histological tumor types studied were mixed carcinoma and simple adenoma. The cells were phenotypically characterized according to an immunocytochemical panel, including cytokeratins, alpha smooth muscle actin and vimentin.

## Results

Results have shown that the best method of obtaining primary epithelial cell lines comprises the use of collagenase type I, hyaloronidase and trypsin followed by serial differential trypsinization.

## Conclusions

Standardization of such methodological tools in the canine model for the study of cancer will allow a more detailed analysis of the action of new antineoplastic agents, which could be applied to animals and, eventually, to humans.

## Financial support

FAPESP and CNPq.

